# The dose makes the poison—Novel insights into Dravet syndrome and *SCN1A* regulation through nonproductive splicing

**DOI:** 10.1371/journal.pgen.1009214

**Published:** 2021-01-07

**Authors:** Ingo Helbig, Ethan Goldberg

**Affiliations:** 1 Division of Neurology, Children’s Hospital of Philadelphia, Philadelphia, Pennsylvania, United States of America; 2 The Epilepsy NeuroGenetics Initiative (ENGIN), Children's Hospital of Philadelphia, Philadelphia, Pennsylvania, United States of America; 3 Department of Biomedical and Health Informatics (DBHi), Children’s Hospital of Philadelphia, Philadelphia, Pennsylvania, United States of America; 4 Department of Neurology, University of Pennsylvania, Philadelphia, Pennsylvania, United States of America; 5 Department of Neuroscience, University of Pennsylvania, Philadelphia, Pennsylvania, United States of America; University of California San Francisco, UNITED STATES

## Dravet syndrome—A paradigmatic developmental and epileptic encephalopathy moving toward precision medicine

In 1978, the causes of most severe childhood epilepsies were entirely unknown, and any ability to systematically apply massive parallel sequencing to understand the underlying etiologies was still in the far future, while targeted therapy for a genetically defined epilepsy was at best a fantasy. During this period, Charlotte Dravet and collaborators first described a distinct epilepsy syndrome characterized by fever-induced seizures, generalized features on electroencephalography, and developmental plateauing starting in the second year of life, a condition initially called Severe Myoclonic Epilepsy of Infancy [1]. Fast forward 40 years and the condition now referred to as Dravet syndrome represents one of the most common developmental and epileptic encephalopathies with an estimated frequency of 1:15,000 [2]. Disease-causing variants in *SCN1A* are identified in up to 80% of individuals with clinical Dravet syndrome, and hundreds of individuals are diagnosed every year. *SCN1A* encodes a voltage-gated sodium channel subunit that is critical for neuronal function, specifically the function of GABAergic interneurons. While Dravet syndrome represent the main clinical phenotype for *SCN1A*-related disorders, milder clinical presentations are known that are typically seen in families [3]. This group of fever-associated epilepsies is usually referred to as Genetic Epilepsy with Febrile Seizures Plus (GEFS+). Developing novel therapeutic strategies for Dravet syndrome represents an active field of research, and novel anti-seizure medications including cannabidiol and fenfluramine were specifically introduced through systematic studies in Dravet syndrome [4,5].

## Poison exons are common in epilepsy genes and represent novel therapeutic targets

Haploinsufficiency is the generally accepted disease mechanism in Dravet syndrome, and until 2 years ago, no strategies were available to affect this primary disease mechanism. Two findings have revolutionized the current understanding of *SCN1A*-related disorders. First, noncoding *de novo* variants in highly conserved intronic regions of *SCN1A* were found in individuals with Dravet syndrome [6]. This conserved intronic region was found to contain a poison exon, and the de novo variants were shown to result in increased poison exon inclusion. In addition, the systematic analysis of *SCN1A* gene expression identified physiological nonproductive splicing events that could subsequently be reduced through specifically designed antisense oligonucleotides (ASO) [7]. This mechanism can be exploited therapeutically, and this strategy is currently implemented through a novel therapeutic method that targets these nonproductive splicing events. This method, referred to as Targeted Augmentation of Nuclear Gene Output (TANGO), resulted in restoration of *SCN1A* expression in a Dravet mouse model with a reduction of seizures and Sudden Unexpected Death in Epilepsy (SUDEP) [7]. This novel technology is currently moving to clinical trials. Nonproductive alternative splicing has also been identified in a total of more than 1,200 disease-associated human genes, including known genes for developmental and epileptic encephalopathies (DEE) such as *SYNGAP1*, *SCN2A*, or *SCN8A* [8]. Therefore, these strategies might result in a broad range of novel therapeutic strategies. The underlying mechanism of these poison exons is intriguing. Alternative exons containing a premature termination codon are extremely common, and with up to 30% of all human genes with poison exons, they appear to be the norm rather than the exception [9]. This raises the question with regards to their physiological function and their role during normal development.

## A mouse model due to an *SCN1A* poison exon mutation replicates features of Dravet syndrome

In the study by Voskobiynyk and colleagues [10], the authors provide a deep dive into regulation of *SCN1A* through poison exons. The *SCN1A* gene contains the naturally occurring poison exon 20N, and inclusion of this exon results in nonproductive splicing and nonsense-mediated RNA decay, effectively reducing the amount of Nav1.1-containing sodium channels made available to neurons. The authors characterize a Dravet syndrome mouse model with a poison exon mutation resulting in aberrant *Scn1a* regulation. The specific disease-causing poison exon variant was previously identified in a patient with Dravet syndrome reported by Carvill and colleagues [6]. Voskobiynyk and colleagues observe premature mortality, seizures, and behavioral findings, suggesting that the mouse model replicated some of the clinical features of Dravet syndrome. This mouse model provides support for the inference that the inclusion of a poison exon is a mechanism of Dravet syndrome and could represent a valuable tool for future attempts to develop methods to manipulate poison exon inclusion/exclusion to modulate Dravet syndrome pathology in a preclinical system. The most fascinating aspect of their publication is the analysis of gene expression in this model system, comparing expression of wild-type exon 20N and exon 20N carrying the disease-causing noncoding variant. While only approximately 1% of *Scn1a* transcripts carry poison exon 20N in wild type, this ratio is increased 5-fold in animals with the poison-exon mutation. This increase in poison exon inclusion alone resulted in a decrease of *Scn1a* mRNA to 50%, resulting in a reduced gene expression known to occur in Dravet syndrome. The presence of poison exons hints at complex underlying regulatory mechanisms that remain unknown to date and that may represent a promising avenue of future research.

## Persistent poison exon inclusion results in aberrant fetal expression patterns

**Fig 1:**
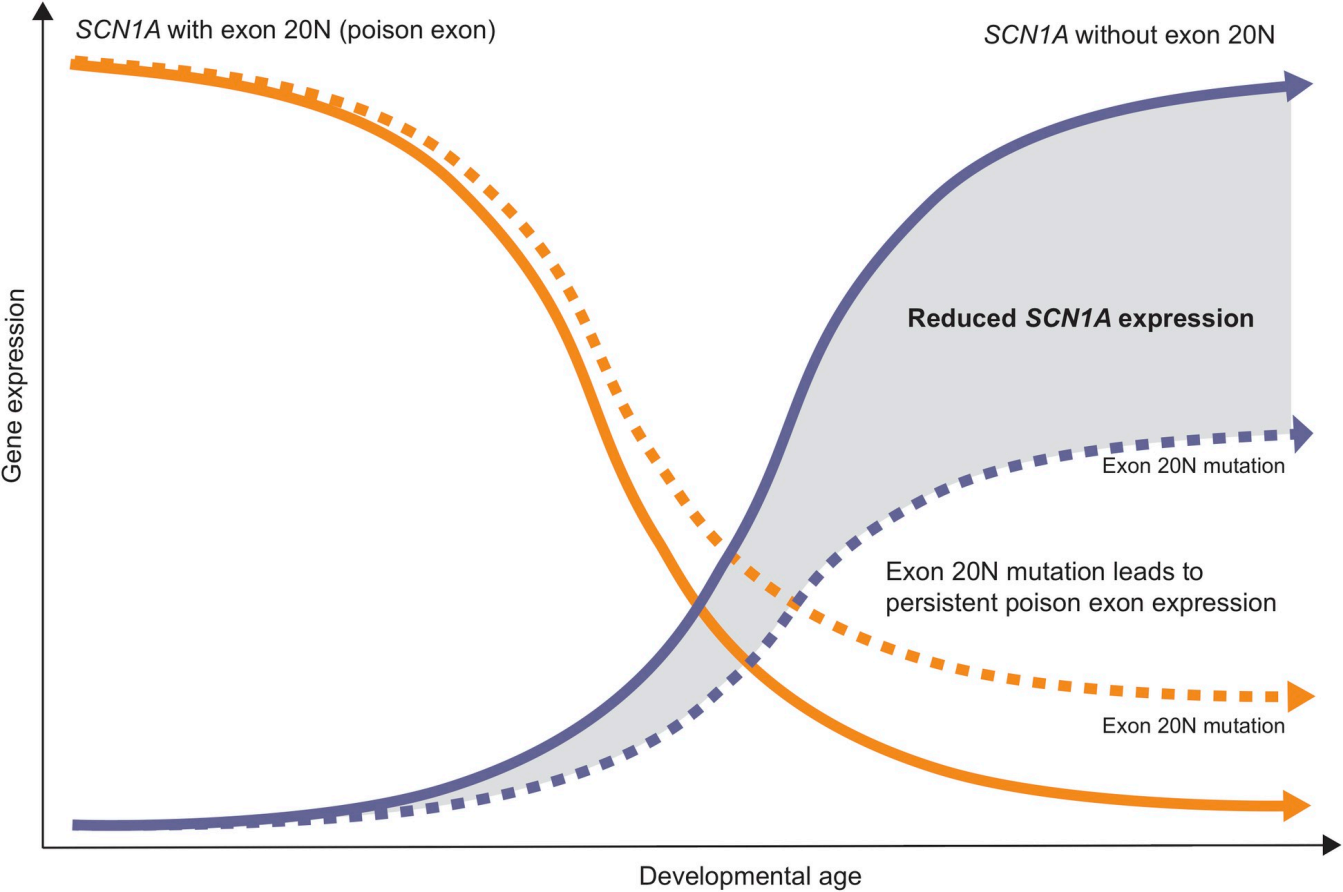
Reduced *Scn1a* expression postnatally due to an exon 20N mutation in a novel mouse model of Dravet syndrome. Introduction of a noncoding *de novo* variant identified in a human patient into a mouse model replicated the phenotype of *SCN1A*-related Dravet syndrome. The reduction of *SCN1A* expression is due to persistent expression of *SCN1A* exon 20N, a “poison exon” resulting in nonproductive splicing. Exon 20N expression is high during embryonal development and leads to suppression of *SCN1A* expression. The noncoding mutation maintains the embryonic pattern of exon 20N expression, resulting in reduction of *SCN1A* transcript.

Voskobiynyk and colleagues [10] then proceed to assess poison exon inclusion and found that up to 70% of *SCN1A* transcripts included exon 20N during embryonal development, which decreased over time to 10% postnatally. This suggests that poison exon inclusion is not just an epiphenomenon, but an active regulatory process to suppress *SCN1A* expression during early development. Poison exon inclusion during normal development then decreases successively postnatally (Fig 1). This allows *SCN1A* expression to be released from the poison exon-mediated shutdown. Therefore, mutations resulting in increased poison exon inclusion do not create an entirely novel transcriptional scenario. Poison exon mutations maintain the embryonal pattern of *SCN1A* regulation, a disease mechanism that could best be referred to as “persistent poison exon inclusion.” In fact, the authors also find a similar pattern of poison exon inclusion for *SCN8A*, a gene also strongly associated with DEE. This indicated that poison exon regulation may be a common feature regulating genes for neurodevelopmental disorders where the precise timing of gene expression is important.

## A paradigm change in genetic epilepsies from genes to transcripts

Where does the study by Voskobiynyk and colleagues leave us? In the past, understanding the basis of genetic causes for epilepsies and neurodevelopmental disorder was considered a question of genomics. Massive parallel sequencing approaches allow up to 30% of DEE to be explained through genetic causes, and the number of known disease genes is increasing steadily. However, as in cancer genomics, it is reasonable to expect a paradigm shift from genes to transcripts. A large number of genes are recognized to contain appreciable amounts of nonproductive alternative splicing through poison exons. Therefore, conceptualizing disease-causing variation as “transcript issues” rather than “gene issues” will enable us to explore the utility of gene regulation approaches in more detail. Many disease-associated genes possess inherent regulatory mechanisms that may provide new targets to restore physiological levels of gene expression. Understanding how these mechanisms change in age-dependent patterns will help identify novel interventional strategies—and the required timing for such interventions—for conditions that are currently still largely treated symptomatically.
